# An End-to-End Deep Learning Approach for Quantitative Microwave Breast Imaging in Real-Time Applications

**DOI:** 10.3390/bioengineering9110651

**Published:** 2022-11-04

**Authors:** Michele Ambrosanio, Stefano Franceschini, Vito Pascazio, Fabio Baselice

**Affiliations:** 1 Dipartimento di Scienze Motorie e del Benessere, University of Napoli Parthenope, Via Medina 40, 80133 Napoli, Italy; 2 Centro Direzionale, Dipartimento di Ingegneria, University of Napoli Parthenope, 80143 Napoli, Italy

**Keywords:** microwave tomography, breast imaging, neural networks, artificial intelligence, electromagnetic inverse scattering

## Abstract

(1) Background: In this paper, an artificial neural network approach for effective and real-time quantitative microwave breast imaging is proposed. It proposes some numerical analyses for the optimization of the network architecture and the improvement of recovery performance and processing time in the microwave breast imaging framework, which represents a fundamental preliminary step for future diagnostic applications. (2) Methods: The methodological analysis of the proposed approach is based on two main aspects: firstly, the definition and generation of a proper database adopted for the training of the neural networks and, secondly, the design and analysis of different neural network architectures. (3) Results: The methodology was tested in noisy numerical scenarios with different values of SNR showing good robustness against noise. The results seem very promising in comparison with conventional nonlinear inverse scattering approaches from a qualitative as well as a quantitative point of view. (4) Conclusion: The use of quantitative microwave imaging and neural networks can represent a valid alternative to (or completion of) modern conventional medical imaging techniques since it is cheaper, safer, fast, and quantitative, thus suitable to assist medical decisions.

## 1. Introduction

### 1.1. Motivation

Breast cancer is one of the commonest types of cancer which affects women, and its early detection is of vital importance for successful treatments [1,2,3]. Among the commonest clinical imaging and diagnostic modalities employed for this aim it is worth mentioning X-ray mammography, magnetic resonance imaging (MRI), ultrasound scanning and nuclear medicine [4].

X-ray mammography is based on the use of very-high-frequency radiations (10 PHz–10 EHz) which travels along the scanned area. Thus, it exploits ionizing radiations and it represents an uncomfortable exam due to compression of the breast to carry out the diagnosis. Some other limitations are related to the limited dynamic range, low contrast and grainy image, for which it is difficult to visualise very subtle lesions in women who have implants or surgical scars [5]. Another disadvantage is related to its poor spatial resolution and the requirement of a large storage place.

Conversely, due to the use of ionizing radiation to perform the investigation, the use of breast MRI is more recommended for women who are high risk, representing a valuable alternative to X-ray mammography since it does not involve any ionizing radiation exposure. This medical exam produces good spatial resolution images but has some limitations related to the low specificity which results in further tests and biopsies, which drives higher costs [6]. Besides being expensive, this imaging modality is not portable, is slow in the acquisition process and is unsuitable for patients with metallic devices implanted.

Breast ultrasound imaging, which is based on the use of ultrasonic mechanical waves for non-invasive diagnostics, is used as a follow-up test for abnormalities found by mammogram and provides some guidelines for biopsy inspections. This medical examination produces both qualitative and quantitative diagnostic information with a good image quality [7]. The main drawbacks are related to the operator-dependent nature of this exam and to the poor resolution of the image and the low contrast.

Complementary information can be inferred from nuclear medicine, whose functional images are based on the molecular properties of the tissues and on the injected radioactive substance. This imaging modality uses ionizing radiation, as in the mammography case, and it is very expensive. Different from the other imaging modalities, it can investigate the physiological function of the system, but has limited resolution and slow imaging time [8].

The drawbacks and limitations of the aforementioned diagnostic methodologies have motivated the research community to develop new imaging techniques and modalities to realise early, reliable and inexpensive diagnostics. In this framework, the use of microwaves for breast imaging and cancer detection has received significant attention, since they might provide better sensitivity and safety due to their non-ionizing nature [9,10].

Microwave tomography exploits microwave signals to investigate breast tissues and provide quantitative permittivity and conductivity maps (the dielectric properties) of the imaged tissues. This imaging technique offers several advantages over other classical methods since it is not expensive, is non-ionizing, and is comfortable with respect to the treatment [11]. The capability of these systems to perform the imaging is related to the fact that different types of tissues, including both normal and malignant ones, have different electrical properties in the microwave frequency range, both in terms of permittivity and conductivity values [12]. Thus, by virtue of these differences, microwave imaging might represent a good candidate to discriminate between healthy and cancer tissues, and the clinical benefits deriving from its use for imaging purposes are supported by several articles in the scientific literature [9,11,13,14,15].

In particular, microwave systems can provide complementary information on the investigated regions which can be merged with the one deriving from traditional exams to support medical decisions. Nevertheless, there are still some technical challenges and open topics to be solved, which involve both hardware and software [11,15]. Among them, it is worth mentioning the challenges related to the choice of an effective coupling medium to maximise the microwave signal transmitted into the body, the design of proper microwave antennas to allow a high enough number of sensors in a small area to investigate the region of interest, and the reduction of the impact of mutual coupling between close antennas and electromagnetic interference with other radio-frequency devices.

Moreover, another main limitation, especially for quantitative microwave imaging, is related to the well-known nonlinearity and ill-posedness issues and their consequences affecting these kinds of inverse problems, such as the presence of local minima [16], and whose difficulty further increases when dealing with three-dimensional modelling, which is computationally demanding. In particular, the impact of the ill-posedness can be partially mitigated by exploiting some a priori information [17,18,19].

It is worth noting that, since the use of microwave imaging in the medical community is relatively recent compared to other state-of-the-art approaches, the authors intended this work to explore the potentialities that the proposed neural-network-based methodology allows us to reach in terms of imaging performance in the context of microwave breast imaging. For these motivations, in this manuscript we did not report a comparison with other conventional imaging modalities nor some recoveries including the malignant-tissue case, which will be properly considered in future work. The main aim of this work is to explore the potential improvement that the proposed methodology allows us to obtain in terms of the quality of the recovery.

### 1.2. Prototypes

Due to the potentialities of the microwave imaging modality, several research groups developed and realised different prototypes in recent years. In [20], the authors propose a 2D imaging system which consists of a circular array of 12 printed monopole antennas with some recoveries related to simplified phantoms that mimic the human tissues. Further work more focused on the radar system hardware is reported in [21,22,23,24]. Complementary to the previous articles, the effort of the research community was also focused on software aspects, such as finding hybrid nonlinear strategies to improve the recovery performance [25] or the improvement in sensitivity of radar-based breast imaging [26]. All the previously mentioned work contributed to the development of microwave breast imaging. As a matter of fact, the last years have been characterised by several clinical trials [27,28,29,30,31,32]. Nevertheless, these systems all share the feature of employing more antennas arranged around the object of interest with more (virtual or real) transmitters and receivers with the aim of producing an image of the objects located in the investigation region. These approaches are also called *tomographic*.

Initial experimental setups consisted of a three-dimensional (3D) prototype for microwave tomography with more transmitters and one receiver which surrounded the imaging domain and were controlled by a motion control system [33]. In this case, the object to be imaged was immersed in deionized water (used as matching medium) in a cylindrical chamber. Other systems followed this setup with an increment in the number of employed antennas [34]. After these preliminary microwave imaging experiences, some clinical prototypes were developed and tested, still preserving the cylindrical arrangement of the sensors around the imaging area [35,36], but employing a different kind of antenna immersed in a saline coupling medium to improve signal penetration inside the tissues. After that, several improvements followed in both hardware and software [37,38,39] which yielded to the latest arrangement of the antennas located in a hemispherical shape to make the exam more comfortable on the patient’s side [40,41].

Nowadays, some of the aforementioned imaging systems have started their active clinical trials, showing the potentialities of this technology for supporting medical decisions [42]. Some results of these trials are summarised in Table 1.

### 1.3. Algorithms

In order to obtain the permittivity and conductivity maps of the imaged tissues, an inversion algorithm can be adopted for solving the electromagnetic inverse scattering (EIS) problem, which is ill-posed and strongly nonlinear in its general formulation [16]. The non-linearity of the considered inverse problem is related to the multiple scattering interactions between points of different tissues and also inside the same tissue, which makes the problem at hand hard to solve and affected by false solutions. These two challenging issues have stimulated different research groups to develop several methods in order to provide tomographic qualitative as well as quantitative images of the biological tissues under test [16,43]. In this framework, the former approaches aim at identifying the pathology within a region without, necessarily, providing an image of the region under test [10,42] while the latter ones aim at producing full maps of the biological tissues in terms of permittivity and conductivity or in terms of labels which are uniquely related to the considered tissues (i.e., segmentation and classification maps).

The possibility of providing quantitative maps of the electromagnetic properties of tissues under test in a non-invasive way can represent a paramount tool for diagnostic purposes. In this context, the capability of tomographic approaches to provide maps of the investigated biological tissues makes them very attractive for clinical applications. Thus, several imaging strategies have been developed over the past thirty years to solve this problem. With regard to the tomographic approaches, three main categories can be identified: qualitative, approximated and quantitative methods [44].

Qualitative methods aim at solving an inverse obstacle problem [45,46,47] by processing the scattered field and providing an estimation of the total tissue extension in the breast, but not its characterisation (i.e., the type of tissue). Approximated methods exploit some approximations of the scattering phenomena in order to allow easy implementation and to keep the computational complexity low. Although they are quite fast, they suffer from some limitations related to the adopted approximated model, as in the case of the well-known Born and Kirchhoff approximations [48]. Another relevant linearised approach proposed in recent years for biomedical imaging purposes is based on the virtual experiments framework [49], which is based on the combination of real and “virtual” experiments that propose a linear approximation of the EIS problem in the case of non-weak scattering regime. Furthermore, higher-order Born approximations can be exploited to reconstruct the conductivity function of the dielectric tissues under test [50].

In order to overcome the limited retrieving performance related to the aforementioned classes, quantitative approaches can be exploited [51]. With regard to these methods, the class of retrievable objects becomes wider at the expense of higher computational burden and processing time. Different iterative approaches can be employed to solve this problem and to face the issues related to non-linearity which may drive into false solutions due to the presence of local minima. Due to the high computational complexity of these methods, some local minimisation approaches are adopted and thus the choice of the initial step is paramount [52]. However, when the non-linearity of the problem at hand is very strong, then it might be beneficial to use global optimization to avoid local minima [44,53]. Unfortunately, their complexity grows exponentially with the number of unknowns and this makes their use very hard to apply for realistic and/or real-time applications. Moreover, due to non-linearity, the inversion procedure may be more sensitive to modelling errors and uncertainties on the scenario.

### 1.4. Machine Learning for Quantitative Microwave Imaging

In the framework of inverse problems, fast and reliable non-linear approaches are desirable for addressing the imaging problem in the biomedical area of breast cancer diagnostics. Among the most recent methodologies, artificial neural networks represent a useful and flexible tool for quantitative imaging. As a matter of fact, neural networks and artificial intelligence proved to perform well in the field of computer vision, image processing and classification. First, methodologies based on artificial neural networks were applied to extract some general information about the geometric and electromagnetic properties of the scatterers and tissues at hand [54,55]. Most of these first attempts to face the imaging problem via neural networks used a few spatial as well as electromagnetic parameters to represent the scatterers.

Recently, most of the literature has focused on the use of deep convolutional neural networks (CNNs) for solving the inverse problem [56,57,58,59,60,61,62]. Neural networks with regression features have provided very impressive results on EIS problems. The majority of these articles do not propose a direct inversion scheme, i.e., the approach does not allow us to move directly from the data collected at receivers to an estimate of the profile, but they usually perform a super-resolution of the recovery starting from a raw image obtained via other conventional approaches. One of the most adopted techniques consists of the training of a U-net architecture [63] for obtaining quantitative recoveries via preliminary manipulations, e.g., approximated models and a priori information to move from the data (i.e., the scattered field samples) to contrast/induced currents approximations [57,58]. In order to solve the EIS problem with high contrast, a contrast-source-based neural network combined with a traditional subspace-based optimisation method and CNNs might be employed [59]. Furthermore, neural networks can be also employed as regularisation strategies in conventional inversion approaches [64,65] as well as to obtain super-resolved reconstructions [66].

Contrary to the contemporary scientific literature which focuses on the use of CNNs, in this work we focused on artificial neural networks (ANNs) based on multilayer perceptrons. This kind of network allows us to implement a direct inversion scheme from the scattered field samples to directly retrieve a quantitative map of the dielectric features of breast profiles in a fast, efficient way. Despite the ease in network design, a critical issue lies in the choice of a large enough dataset for training the network, which proves to be of vital importance for the estimation of the links’ strength between nodes [63]. Thus, the main bottleneck of this kind of approaches is related to the computational burden required to train the network. Nevertheless, after the initial training, then a direct mapping between data and unknowns can be obtained, producing reliable images in a considerably fast inversion procedure.

Inspired by the universal approximation theorem (UAT) [67]—which states that any arbitrary non-linear function can be approximated via a proper fully-connected neural network with a large number of neurons in its hidden layers under some mild assumptions—in this manuscript we propose an ANN architecture for the real-time *quantitative* imaging of female breast dielectric properties. The motivation in choosing such an architecture is supported by the need to define a general approach for the retrieval of whatever non-linear profile, as supported by the UAT. As a matter of fact, in an ANN architecture, all the inputs contribute to every single output, resulting in being more suitable for this kind of application than CNNs, the latter being the default choice when dealing with highly-structured modalities such as images or video.

It is worth noting that the proposed work is focused on the potentialities that deep learning via quantitative tomographic imaging at microwave frequencies can deliver in the framework of biomedical breast imaging. Moreover, the use of an end-to-end network which implements a direct inversion scheme further strengthens the proposed approach, allowing the processing of the scattered field samples to provide a quantitative map of the tissues properties.

Therefore, in short, it is possible to summarize the main element of novelty of the considered work into three main aspects:

- The use of *fully-connected* neural networks to perform quantitative imaging of the *breast tissues*;
- The use of a *direct* inversion scheme to obtain the permittivity and conductivity maps of *breast tissues*;
- The realistic *in-house* numerical phantom generator and the corresponding dataset for an overall population of 120,000 elements, which is paramount for a proper training of the neural networks to perform a certain task, and therefore an important element of novelty.

On the other hand, the main limitations of the proposed methodology, as with most deep learning approaches, lie in the initial training time necessary for the correct operation of the neural networks and in the database generation which is task-specific. Nevertheless, it is worth noting that, except for the initial training time, the quantitative imaging of the target tissues is very fast (less than 0.01 s on a Linux Mint machine with an AMD Ryzen Threadripper 3990X processor and 250 GB RAM memory).

The outline of the paper is as follows. In Section 2, an overview of the mathematics involved in electromagnetic inverse scattering (EIS) problem is recapped. In Section 3, an overview of the proposed fully-connected ANN approach is reported with a focus on the dataset generation dealing with a realistic breast-like phantom generator for the database population. Finally, some results on synthetic breast phantoms are shown in Section 4 and a comparison with conventional nonlinear EIS approaches is proposed. Some conclusions are drawn at the end of the article.

## 2. Problem Statement

In the following, a simplified two-dimensional geometry is considered. The background medium is supposed to be homogeneous with complex permittivity $\epsilon_{b}$ and with magnetic permeability $\mu_{0}=4\pi \cdot 10^{-7}$ H/m. The reason for such an assumption is related to the fact that biological tissues are characterised by a constant value of the magnetic permeability while a certain variability of the complex permittivity can be observed. The antennas are located along a measurement curve Γ which surrounds the imaging domain Ω. The targets located inside this domain are illuminated via transverse-magnetic electric fields generated by z-oriented current wires located on Γ.

**Figure 1:**
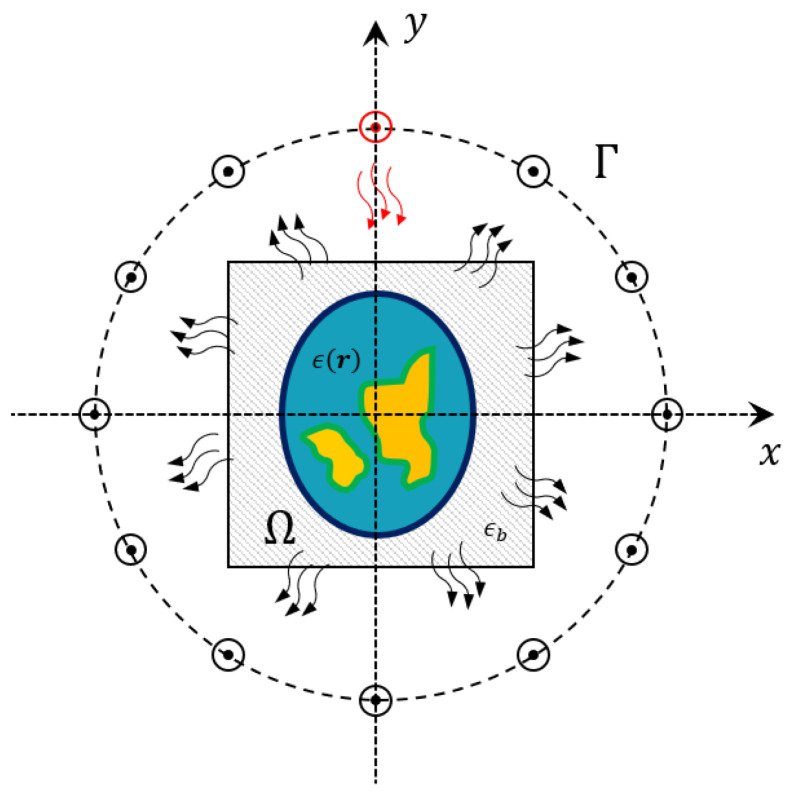
Sketch of the 2D geometry. The antennas are located on the measurement line Γ and arranged in a multiview-multistatic fashion.

A sketch of the geometry is reported in Figure 1. The scattering phenomena depend on the contrast between the dielectric properties of the background medium and those ones of the target. The aim of the proposed method is to provide the breast tissue properties maps by solving an EIS problem, i.e., their relative complex permittivity $\epsilon \left(r\right)=\epsilon^{\prime }\left(r\right)-j\frac{\sigma \left(r\right)}{\omega \epsilon_{0}}$, with $\epsilon^{\prime }\left(r\right)$ and $\sigma \left(r\right)$ being the relative permittivity and conductivity maps, respectively. Thus, the electromagnetic scattering equations ruling these phenomena can be written as [16]:

(1) $$\begin{array}{cc} E_{s} & =A_{e}\left(\epsilon^{\prime },\sigma ,E_{t}\right)+n\;, \end{array}$$

(2) $$\begin{array}{cc} E_{t} & =E_{i}+A_{i}\left(\epsilon^{\prime },\sigma ,E_{t}\right)\;, \end{array}$$

in which the dependence on the operating frequency and background dielectric features have been implied. $E_{i}$ and $E_{t}$ represent the incident and total electric field inside the imaging domain Ω, respectively, while $E_{s}$ represents scattered field at receivers locations on Γ. The quantities $A_{i}$ and $A_{e}$ are the radiating operators which depend on the dielectric properties of background medium $\epsilon_{b}$, and *n* is the noise which affects the collected data, here assumed to be additive, white and Gaussian (AWGN).

As regards the imaging applications, the considered framework can be dealt with as an EIS problem consisting of the retrieval of a quantitative estimate of the unknown relative permittivity $\epsilon^{\prime }$ and conductivity σ functions inside Ω from the scattered field samples measured on Γ. As previously stated in Section 1.3, such a problem is both nonlinear and ill-posed [43,68,69], thus finding a solution to this problem is not trivial and requires facing a high computational burden and time-consuming approaches. Nevertheless, reliable and fast algorithms able to provide dielectric properties maps of the tissues under test are desirable for early diagnosis in the biomedical field. In this framework, conventional nonlinear approaches can achieve good recovery performance [70,71,72,73,74] at the expense of high computational burden, which might imply no real time applications. Moreover, considerable a priori information and data pre-processing are required to obtain reliable recoveries.

In this framework, artificial neural networks (ANNs) can be of interest and represent a very attractive alternative to real-time applications with more accurate reconstructions. In this case, the challenge is related to the network design and the dataset generation for the training step which considerably impacts on the recovery performance.

## 3. Methodology

In order to define the proposed approach to solving the inverse problem at hand, two main aspects have to be described: the characteristics of the database adopted for the ANN training and the layout of the implemented neural network.

### 3.1. Breast Database

**Figure 2:**
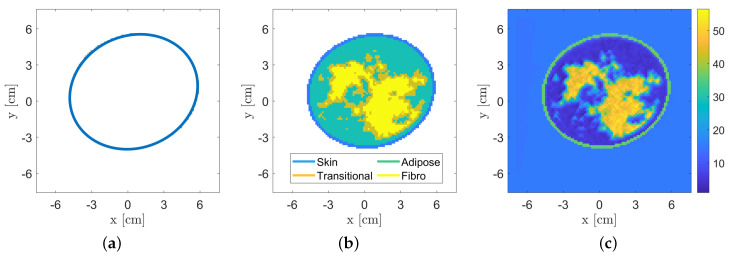
Overview of the realistic numerical breast phantom generation procedure. (**a**) Elliptical-shape phantom generation with corresponding skin layer. (**b**) Internal-tissue spatial distribution via a random field generator [77] and (**c**) estimated electromagnetic (i.e., relative permittivity and conductivity) tissues assignment (for the sake of brevity, only the relative permittivity is reported).

In order to perform quantitative inversion via neural networks in a reliable way, it is fundamental to properly model the specific scenario under test and to have large datasets of realistic breast profiles. Some research groups focused on the generation of these datasets to be used by the scientific community [75,76]. Unfortunately, in most cases the available population is very limited or not flexible enough to be exploited in the framework of neural networks training, since they are limited to a specific measurement configuration and/or in a certain frequency range. In order to overcome these limitations and with the aim of providing a useful simulation tool for the testing of breast microwave imaging algorithms, a numerical realistic breast phantom generator has been developed to populate the database. After that, the corresponding scattering matrices are calculated, exploiting a forward solver based on the method of moments (MoM). These matrices, together with the generated breast profiles, represent the data adopted for the proposed neural network approach. Figure 2 illustrates the main steps of the breasts’ phantom generation, which together with the related scattering matrices define the database. The whole procedure is detailed in the following.

It is worth noting that the aim of the generated dataset is focused on providing a performance assessment of artificial neural networks (ANN) approaches for quantitative microwave breast imaging to be used for clinical applications of breast cancer. Future work will deal with the improvement of recovery performance, tumor detection and characterization at different operating conditions.

The first step of the generator consists of the creation of an elliptical-shape phantom. The dimensions of the ellipse axes vary uniformly in the range [6.5,12] cm. The center of the ellipse is randomly set inside a circle of radius 1 cm and located in the middle of the imaging domain. The orientation of the ellipse and its thickness, emulating the skin layer, are also randomly selected in the range [0,2π] and [1.5,2.5] mm, respectively.

The geometry of breast inner tissues is generated by exploiting a stochastic 2D multi-fractal random field generator [77]. The obtained map is segmented into three tissues, i.e., the *fibro-glandular*, the *transitional* and the *adipose* tissues. Similar to [12], we chose to simulate four breast classes characterized by different tissues percentage, as reported in Table 2, to test the inversion performance.

In order to generate realistic values of the tissues dielectric features, they are generated according to the statistical distributions estimated from the database described in [12]. Finally, spatial correlation among neighbouring pixels has been added to the data. This procedure is repeated to populate the database composed of 120,000 profiles (30,000 per each breast class). For each profile (discretized into 108×108 pixels), the scattering matrix has been computed via a fast Fourier transform-conjugate gradient (FFT-CG) forward solver based on the MoM [68] assuming a multiview-multistatic system with transmitters and receivers located in 30 angular equally-spaced locations on a measurement circle of radius 12 cm. The transmitting signal is a line source at a fixed frequency which impinges on an imaging domain of size 15×15 cm^2^ discretized according to Richmond’s rule [78].

The database has been partitioned in order to use the 85% of the profiles for the training phase, the 10% for the validation phase and the remaining 5% for the testing.

### 3.2. Neural Network Design and Training

Differently from other approaches presented in literature, in this manuscript a fully-connected architecture is considered instead of the commonly adopted CNNs. Some preliminary (and partial) work can be found in [79,80,81].

The key concept of an EIS inversion consists of the fact that a direct mapping between two different spaces, i.e., the scattered field and the dielectric contrast, is established. Most of the work proposed in the scientific literature focuses on the use of CNNs architecture [56,57,58], but these architectures are very useful typically when the input information flow is local, i.e., each output value is related to an input subset, such as denoising or despeckling applications [82,83]. Conversely, in the case of EIS problems, there is not a direct link between the data and unknown spaces, therefore a preliminary extra-mapping is usually required for reaching good accuracy and optimal inversion performance. Practically, an initial raw inversion is mostly performed to move from the data to the unknown space, and then a CNN architecture is usually applied which represents the second part of a two-step inversion procedure [58]. Thus, for imaging purposes, the fully connected ANN’s feature of having global links between all nodes of consecutive layers seems to be the key aspect to us.

**Figure 3:**
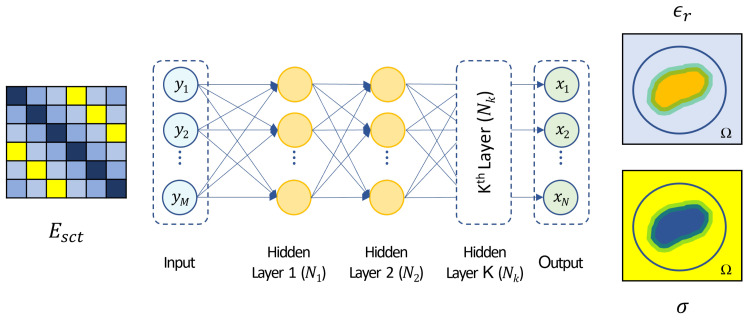
Sketch of the inversion procedure. The input of the fully-connected neural network is the scattering matrix $E_{sct}$ re-arranged in a vector form $y_{i},\;i=\left\{1,2,\cdots ,M\right\}$, with *M* number of scattered field samples. The output of is represented by the tissues relative permittivity $\epsilon_{r}$ and conductivity σ pixel by pixel $x_{j},\;j=\left\{1,2,\cdots ,N\right\}$, with *N* number of pixels in the imaging domain.

A sketch of the adopted neural network architectures is reported in Figure 3. As previously mentioned, a direct mapping between the scattered field samples ($y_{i},\;i=\left\{1,2,\cdots ,M\right\}$, with *M* number of scattered field samples) and the tissues relative permittivity $\epsilon_{r}$ and conductivity σ pixel by pixel ($x_{j},\;j=\left\{1,2,\cdots ,N\right\}$, with *N* number of pixels in the imaging domain) is established. In the network architectures considered in the following, an adaptive moment estimation method (ADAM) was employed to minimize an $l^{2}$-cost function with an initial learning rate of $5\cdot 10^{-5}$ and 30 epochs per each training phase. It is worth noting that in all the considered numerical tests, a wide enough dataset (composed of 102,000 breast profiles for the training) was adopted to avoid the risk of overfitting, as confirmed by the validation and training loss curves. This aspect, which also drives a heavy computational burden due to the high number of trainable parameters to be learnt, represents one of the main limiting factors compared to convolutional neural networks and other conventional approaches. All the information mentioned previously regarding the adopted neural network architecture and its training is summarized in Table 3 for the sake of clarity.

A proper numerical analysis of the optimal network layout in terms of number of hidden layers and neurons is reported in the performance assessment in Section 4.1. Regarding the computational time, the training of each architecture depends on the number of involved layers and varies from 3 h (1 layer) up to 8 h (5 layers), while the inversion phase is real-time, requiring about 3.5 ms to estimate the permittivity and conductivity maps. These evaluations were performed on a Linux 64 bit workstation with an AMD Ryzen 3990X processor and an NVIDIA Quadro RTX 6000 graphics card.

### 3.3. Quality Performance Indicator on Testing Population

In order to perform a numerical assessment, some quantitative, fair metrics to determine the quality of the recoveries are required. Conventionally in the image processing community, parameters such as the normalized root mean square error (NRMSE) and the structural similarity index measure (SSIM) are considered. Nevertheless, every metric has its own peculiarities and makes it possible to capture only a limited amount of information regarding the retrieved image. Thus, in order to integrate this information and to evaluate the capability of the approach in terms of details retrieval, another quality measure based on the analysis of the spectral features of the reconstruction profile is here proposed. This approach can be considered as the evaluation of the filtering properties of the inverse radiating operator and of the imaging algorithm.

For every image of the testing set, the 2D power spectrum in polar coordinates (ν,θ) was evaluated and averaged with respect to all the images spectra, obtaining a mean-squared spectrum T(ν,θ). By averaging along the angle coordinate, a one-dimensional spectrum S(ν) is obtained, i.e.,

(3) $$\begin{array}{cc} \; & T\left(\nu ,\theta \right)=\frac{1}{N}\sum_{n=1}^{N}\left|F({\tilde{x}}_{n}\right){|}^{2}\;, \end{array}$$

(4) $$\begin{array}{cc} \; & S\left(\nu \right)=\frac{1}{2\pi }\int_{-\pi }^{\pi }T\left(\nu ,\theta \right)d\theta \;, \end{array}$$

in which N=6000 is the number of testing profiles, F is the Fourier transform operator, ${\tilde{x}}_{n}$ is the retrieved estimate of the breast profile obtained via ANN inversion.

The motivation supporting this performance indicator lies in the fact that the closer the recovery is to the true profile, the closer their spectral information will be, providing an indirect measure of the filtering properties of the inverse radiating operator and of its impact at each frequency. This procedure clearly yields a spectral comparison rather than a direct similarity measure. Thus, the radial spectrum indicator of Equation (4) is exploited to further improve the reconstruction performance analysis of the different ANN architectures considered in Section 4.1 and to properly identify a good trade-off between computational burden (for the network training) and high accuracy values.

## 4. Results

In this section a numerical analysis to properly design the network architecture, i.e., the number of nodes and layers, will be addressed. Moreover, the methodology is tested in the case of different scenarios. The recovery performance was evaluated by comparing the proposed ANN reconstructions with those ones obtained via conventional nonlinear approaches.

### 4.1. Performance Assessment

**Figure 4:**
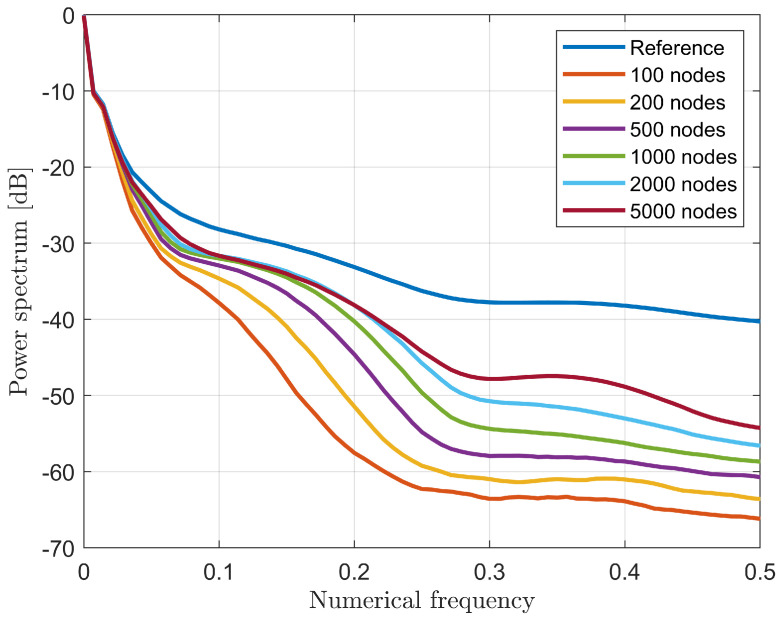
Averaged spectrum S(ν) for six different three-layer architectures as a function of nodes number per each hidden layer.

In order to find a trade-off between computational complexity and accuracy in the recovery, a performance assessment on different network topologies was carried out. As a first analysis, the impact of the number of nodes was evaluated in the case of a three-layer fully-connected network. As expected, Figure 4 shows that the higher the number of nodes, the better the quality of the recovery since the averaged spectrum S(ν) tends to the ideal behavior of the true profile. Nevertheless, the higher the number of nodes, the higher the computational burden, which yields a longer training time. Thus, the 2000-node case was selected since the improvement in the averaged spectrum trend starts to be negligible from this topology and represents a good trade-off for the computational complexity.

**Figure 5:**
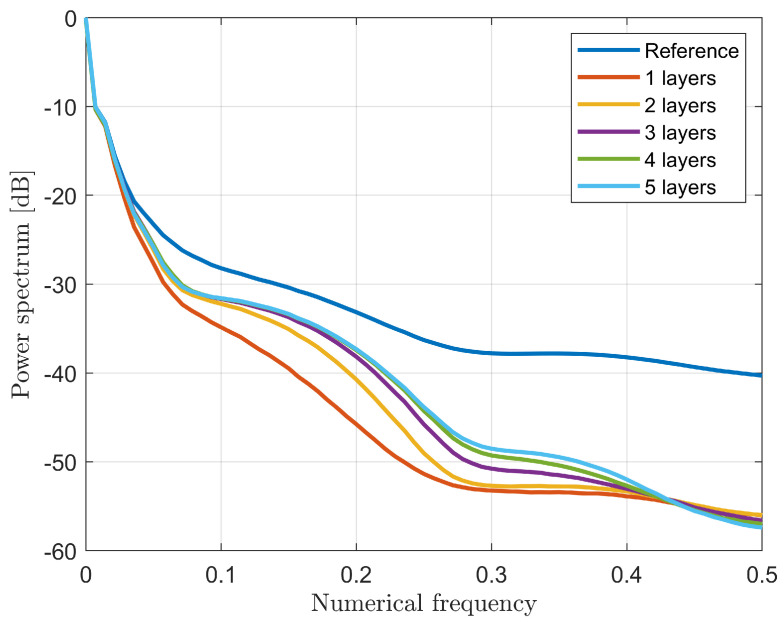
Averaged spectrum S(ν) for five different architectures with two-thousand nodes per layer as a function of the number of hidden layers.

Complementary to the previous analysis, a similar study about the number of layers was performed. To this aim, the averaged spectrum S(ν) is reported while increasing the number of hidden layers from one to five and adopting 2000 nodes per each layer. The related results are shown in Figure 5. In this case, there is no visible difference in the recovery performance among the networks using more than three layers.

Thus, in order to keep the computational burden reasonably low and, at the same time, a reconstruction quality sufficiently high, a three-layer, 2000-node architecture was selected (except the last regression layer not considered in these analyses). It is worth noting that we selected a number of breast profiles in the dataset high enough for ensuring an effective training for all the architectures considered in this work.

### 4.2. Comparison with Other Nonlinear Approaches

The performance of the real-time ANN approach proposed has previously been compared with other well-known nonlinear methods, which are the distorted Born iterative method (DBIM) [84,85] and the contrast source inversion (CSI) [86]. In the former case, a solution to the considered inverse problem is sought via a series of linear problems which gradually recover the full nonlinear profile, while in the latter case an iterative inversion scheme based on a functional minimisation involving both Equations (1) and (2) is exploited. These conventional nonlinear inversion approaches are iterative and usually employ local minimisation schemes in order to avoid prohibitive computational time required by global optimisation schemes. Under these assumptions, the choice of the initial guess for starting the minimization procedure becomes paramount for obtaining good recoveries. In more detail, the authors adopted the DBIM implementation proposed in [70], which is a multi-frequency, multi-threshold sparse-based approach named AMTISTA, and, for the CSI implementation, the one proposed in [87] in its cross-correlated formulation (CC-CSI).

**Figure 6:**
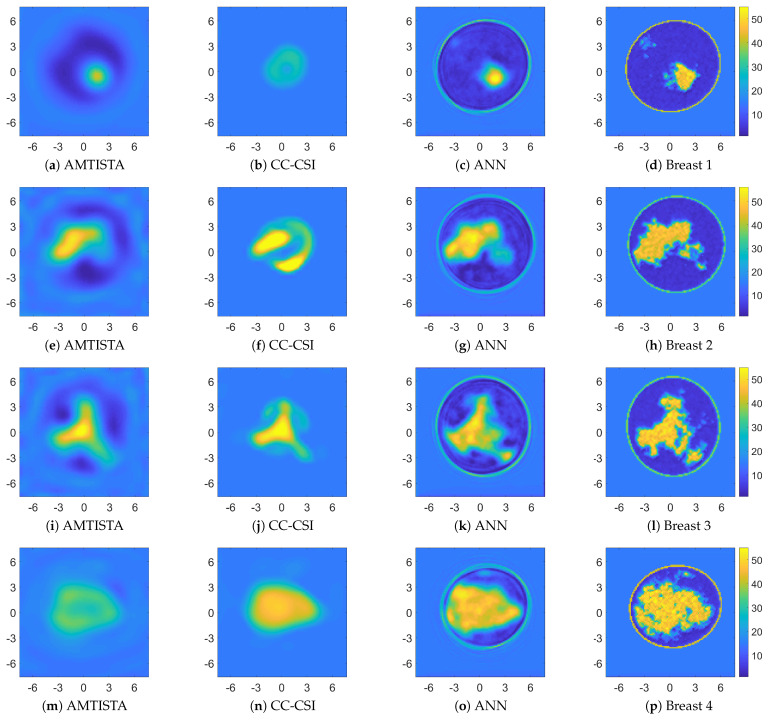
Comparison between recoveries related to four different breast profiles (one per each class). Retrieved relative permittivity maps (dimensionless quantity) for AMTISTA (DBIM formulation as reported in [70]) (**a**,**e**,**i**,**m**), cross-correlated CSI (CC-CSI) [87] (**b**,**f**,**j**,**n**), and proposed ANN approach (**c**,**g**,**k**,**o**). Reference permittivity maps are reported in (**d**,**h**,**l**,**p**). Both the x and y axes are in centimeter units.

**Figure 7:**
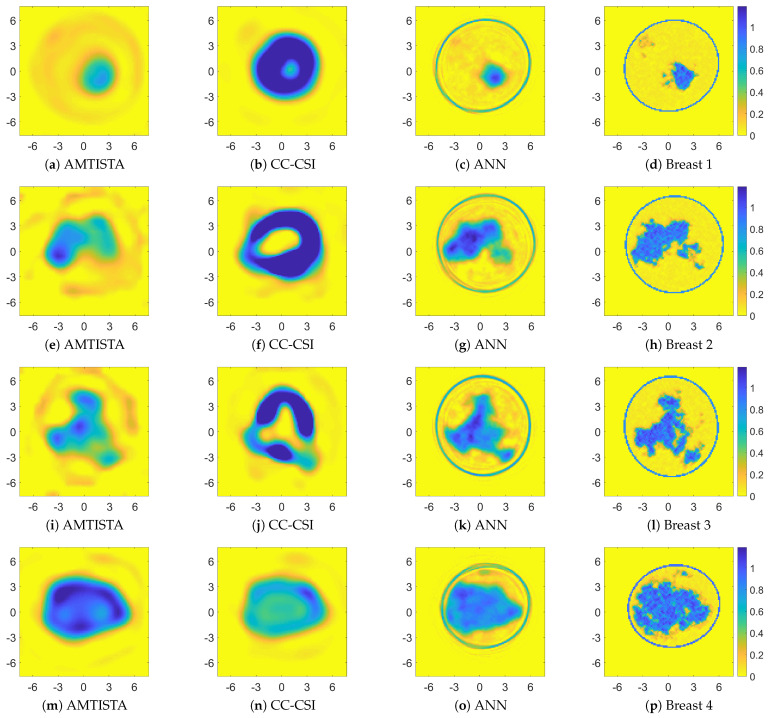
Comparison between recoveries related to four different breast profiles (one per each class). Retrieved conductivity maps (measure unit: S/m) for AMTISTA (DBIM formulation as reported in [70]) (**a**,**e**,**i**,**m**), cross-correlated CSI (CC-CSI) [87] (**b**,**f**,**j**,**n**), and proposed ANN approach (**c**,**g**,**k**,**o**). Reference conductivity maps are reported in (**d**,**h**,**l**,**p**). Both the x and y axes are in centimeter units.

Figure 6 and Figure 7 show the retrieved complex permittivity maps (i.e., real and imaginary parts) for four different breast profiles, one per each breast class (A-D) at the operating frequency of 1 GHz and with data corrupted by additive white Gaussian noise (AWGN) with a signal-to-noise ratio (SNR) equal to 30 dB. Further information regarding the considered configuration and the data generation is reported in Section 3.1. More specifically, these figures compare the recoveries obtained by the proposed ANN approach with other conventional nonlinear inversion strategies. It is worth noting that the recoveries obtained via adopting AMTISTA and CC-CSI were carried out with a frequency-hopping process using five frequencies—{200, 400, 600, 800, 1000} MHz—and starting the initial local minimization procedure from the background. Conversely, the proposed neural network approach processes the single-frequency data with no a priori information and in a direct fashion, i.e., no iteration is required, and it is still able to guarantee good accuracy and a better resolution performance compared with the considered conventional nonlinear approaches as reported in Figure 6 and Figure 7.

**Figure 8:**
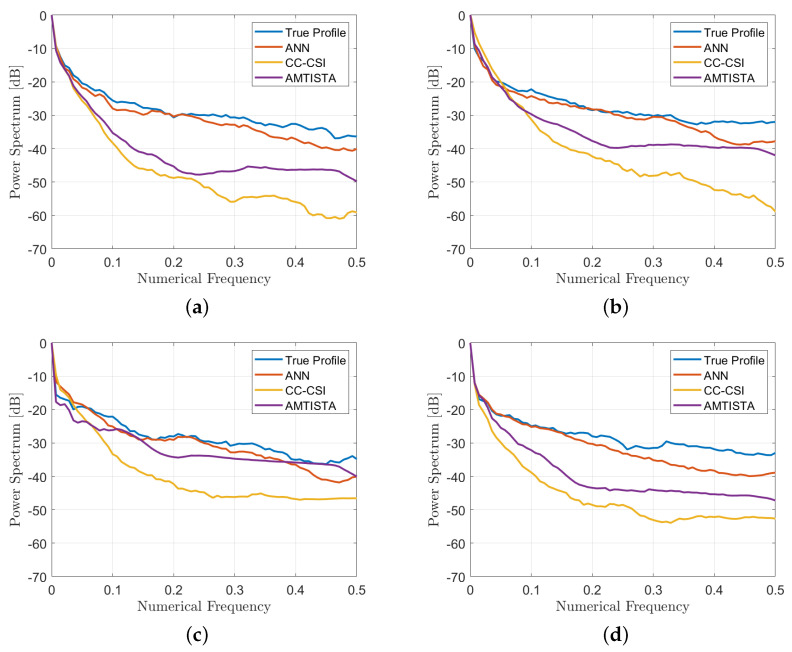
One-dimensional power spectrum indicator of Equation (4) for the retrieved profiles reported in Figure 6 and Figure 7.

To realize a quantitative comparison among the considered reconstructions, three quality metrics were employed further to the quality spectral indicator reported in Equation (4): the structural similarity index (SSIM, values in the range [0, 1], higher is better), the normalised root mean square error (NRMSE, values in the range [0, 1], lower is better) and the correlation coefficient (CORR, values in amplitude in the range [0, 1], higher is better). These metrics were evaluated via default Matlab^®^ functions and are reported in Table 4, while the spectral indicators of Equation (4) are shown in Figure 8.

By comparing the recoveries and looking at the quality metrics, which are better in the ANN case than in the AMTISTA and CC-CSI cases, it is clear that the proposed ANN approach outperforms these classical techniques, providing good performance also on the conductivity estimate, which represents the hardest part to be retrieved by conventional methods and also the most important information for diagnostic and therapeutic treatments.

**Figure 9:**
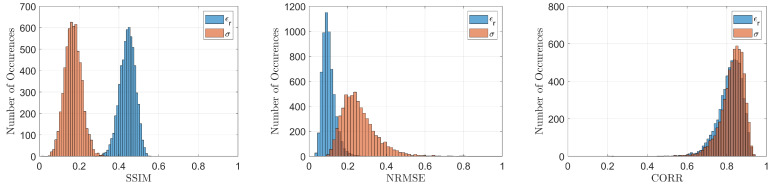
Histograms evaluated on the whole testing population for the adopted metrics.

As further proof of the good and stable recovery performance obtained by exploiting the proposed ANN architecture, Figure 9 and Table 5 report the histograms and corresponding means and standard deviations of the considered quality metrics for the whole testing population. It is interesting to note by considering these values that the recovery performance remains quite stable on the whole testing population regardless of the breast type.

Concerning the computational time, the proposed ANN approach results in a real-time procedure, conversely from conventional nonlinear approaches which are time-consuming per frequency and, sometimes, also per iteration (e.g., the AMTISTA case, for which a forward solver has to be run at each inversion iteration, involving at least 1.5-h simulation for 30 iterations for all the five frequencies on the same workstation). It is worth observing that the proposed ANN approach is able to provide information about the shape and location of the breast and, more specifically, about the skin layer, which still represents an issue for its dielectric features and thickness at the considered operating frequency.

Furthermore, the good recovery performance noticeable in the reconstructions related to tissues relative permittivity maps are also evident in the corresponding conductivity maps, which still represents the hardest, most challenging part in nowadays inverse scattering imaging approaches.

**Figure 10:**
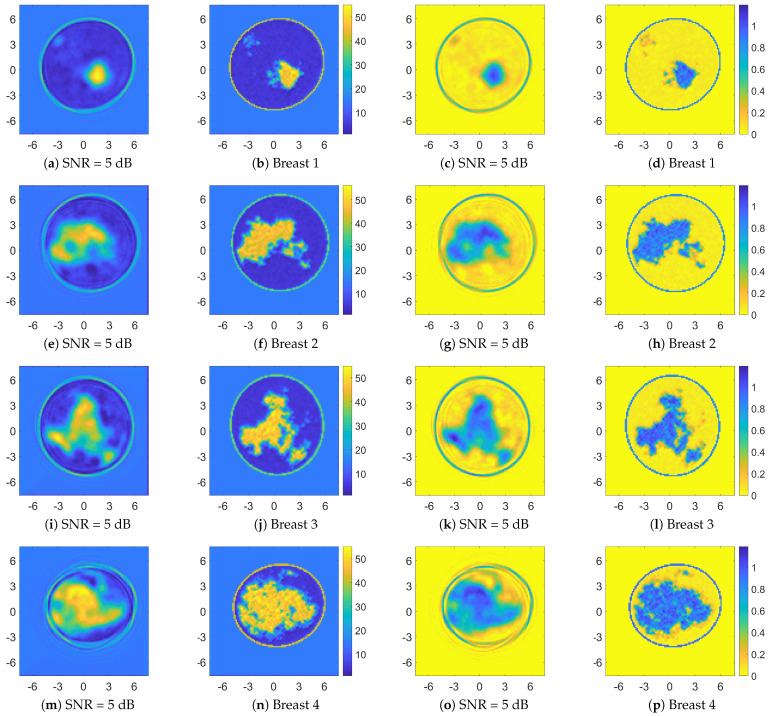
Performance assessment of the proposed ANN approach for an SNR = 5 dB. Retrieved permittivity ((**a**,**e**,**i**,**m**)—dimensionless quantity) and conductivity ((**c**,**g**,**k**,**o**)—measure unit: S/m) maps, and corresponding reference profiles (**b**,**f**,**j**,**n**,**d**,**h**,**l**,**p**). Both the x and y axes are in centimeter units.

In order to test the robustness of the proposed method versus noise, Figure 10 illustrates a comparison among the recoveries of the selected breast phantoms reported in Figure 6 and Figure 7 for a much lower value of the SNR, i.e., 5 dB. The corresponding quality metrics are reported in Table 6. These results prove that the proposed approach is robust against noise and has good inversion performance in very noisy scenarios.

To further prove the robustness of the approach, its recovery performance was tested on realistic numerical breast phantoms generated from magnetic resonance images of female breasts collected by the Cross-disciplinary Electromagnetics Laboratory at the University of Wisconsin-Madison (CEM-UW) [12,75].

**Figure 11:**
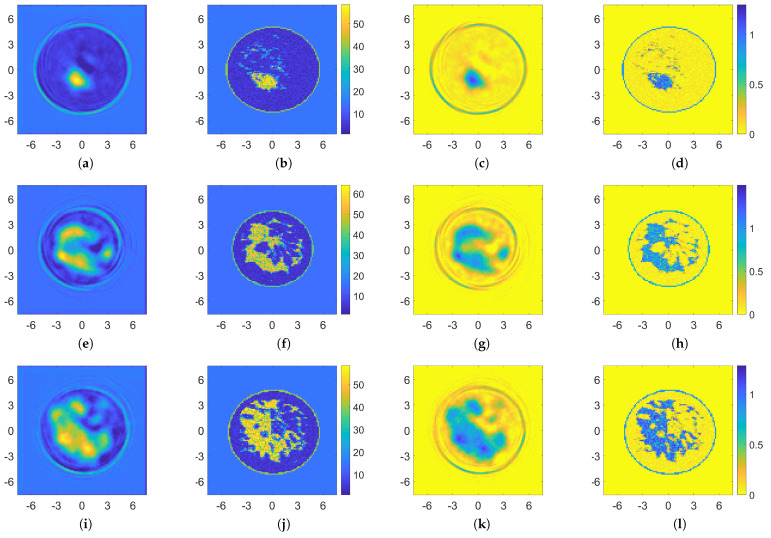
Recoveries obtained via the proposed ANN approach for some realistic breast phantoms proposed by [12,75]. Breast ID: 012204, slice 54: retrieved permittivity and conductivity maps (**a**,**c**) and reference (**b**,**d**). Breast ID: 062204, slices 47 and 71: retrieved permittivity and conductivity maps (**e**,**i**,**g**,**k**), and references (**f**,**j**,**h**,**l**), respectively.

Thus, a few slices of these breast phantoms reported in the repository were considered, i.e., breast ID: 012204, slice 54, and breast ID: 062204, slices 47 and 71, which correspond to some examples of classes B and C, respectively. The measurement configuration and frequency were the same as the ones reported in Section 3.1 and an AWGN noise with SNR = 30 dB was applied to the data. As proved by the results shown in Figure 11, the recoveries were still good, especially on the conductivity of the considered breast profiles, and also allows good enough resolution of the skin layer and a fast inversion approach.

## 5. Conclusions

In this paper, a fully-connected artificial neural network for real-time microwave breast imaging applications has been proposed. The considered network was trained by exploiting an in-house realistic breast-like phantom generator adopted for the dataset population and by in-house forward solver codes for the generation of the scattered field data matrices to be exploited in the training of the network.

The methodology was tested in numerical noisy scenarios with different values of SNR proving good robustness against noise (i.e., 30 and 5 dB) and a performance assessment for the choice of the network architecture was carried out. It is worth noting that the recovery performance depends on the network architecture and each topology provides an improvement in retrieving information in proper bandwidths of the scenario under test.

The results seem good in comparison with conventional nonlinear inverse scattering approaches from a quantitative point of view as confirmed by the metrics reported in Table 4 and Table 5. Indeed, by considering the quality metrics reported in Table 4, it is worth noting that the proposed neural network-based approach allows us to obtain a better recovery performance compared with the considered state-of-the-art approaches in terms of permittivity and conductivity. Furthermore, the values of the metrics reported in Table 5 confirm the general improvement in the quality of the recoveries obtained by the proposed approach on the whole testing population. Lastly, the considered ANN approach proved to be robust versus noise and promising enough to be used on realistic anthropomorphic breast phantoms and, potentially, on experimental data.

The proposed real-time methodology allows us to obtain good recoveries both in terms of permittivity and conductivity maps, the latter usually representing an issue in inverse scattering approaches but also important, useful information for biomedical applications in diagnostics and therapy.

Future work will focus on the design of advanced neural network strategies to improve the quality of the results, enhancing resolution capability, and to process a three-dimensional dataset for the detection and characterization of potential malignant areas, also via exploiting more complex architecture and testing on laboratory experimental data.

## Figures and Tables

**Table 1:** Summary of main microwave breast imaging systems with studies on patients [42].

System	Position	Processing	Frequency [GHz]	Antenna	Scan Time	Reference
Bristol University	prone	radar	3–8	slot	30 s	[26]
Dartmouth College	prone	tomographic	0.7–1.7	monopole	5 min	[22]
ETRI (Korea)	prone	tomographic	3–6	monopole	15 s/slice	[28]
McGill University—table	prone	radar	2–4	TWTLTLA	18 min	[29]
McGill University—wearable	wearable	radar	2–4	microstrip	5 min	[29]
Southern University of China	prone	radar	4–8	horn	4 min	[30]
Calgary University	prone	radar	1.3–7.6	vivaldi	30 min	[31]
Microwave Vision	prone	radar	1–4	vivaldi	10 min	[23]
Mammowave	prone	Huygens	1–9	PulsON P200	10 min	[32]
Kobe University	supine	tomographic	0.05–12	UWB	30 min	[24]

**Table 2:** Breast phantom tissues composition.

Class	Percentage of Tissue (%)		
	Fibro-glandular	Transitional	Adipose
A	5–20	5–15	65–90
B	20–30	10–20	50–70
C	30–40	15–20	40–55
D	40–65	20–25	10–40

**Table 3:** Details on the implemented neural network architecture.

Neural Network Details	
**Input size**	1800 $\left(30\times 30\times 2\right)$
**Output size**	23,328 $\left(108\times 108\times 2\right)$
**Optimization method**	ADAM, initial learning rate: $5\cdot 10^{-5}$
**Training epochs**	30
**Mini-batch size**	64
**Architecture**	3 layers, 2000 nodes per each
**Population**	120,000 (training: 85%, validation: 10%, testing: 5%)

**Table 4:** Quality metrics for both permittivity and conductivity retrieved maps of Figure 6 and Figure 7. The bold quantities refer to the best value per row, identifying the approach which provides the best recovery according to the considered metric.

Breast Phantom	Quality Metric	AMTISTA		CC-CSI		ANN	
		$\epsilon^{\prime }$	σ	$\epsilon^{\prime }$	σ	$\epsilon^{\prime }$	σ
Breast 1	SSIM	0.10	0.02	0.20	0.16	**0.45**	**0.24**
	NRMSE	0.15	0.62	0.35	7.00	**0.06**	**0.22**
	CORR	0.70	0.56	0.06	0.23	**0.89**	**0.86**
Breast 2	SSIM	0.05	0.22	0.17	**0.24**	**0.46**	0.21
	NRMSE	0.14	0.36	0.35	3.44	**0.10**	**0.21**
	CORR	0.78	0.75	0.44	0.18	**0.85**	**0.86**
Breast 3	SSIM	0.06	0.22	0.19	0.18	**0.45**	**0.27**
	NRMSE	0.14	0.36	0.19	1.70	**0.08**	**0.17**
	CORR	0.76	0.74	0.66	0.17	**0.86**	**0.89**
Breast 4	SSIM	0.06	0.04	0.25	**0.30**	**0.40**	0.21
	NRMSE	0.14	0.46	0.10	0.34	**0.07**	**0.17**
	CORR	0.79	0.79	0.81	0.74	**0.87**	**0.88**

**Table 5:** Means and standard deviations of the considered quality metrics per each class of the testing population (6000 breast profiles in total). The number of profiles per class is reported in brackets).

Breast Class	Quality Metrics	Retrieved Permittivity		Retrieved Conductivity	
		Mean	Standard Deviation	Mean	Standard Deviation
A (1422)	SSIM	0.458	0.035	0.182	0.040
	NRMSE	0.102	0.031	0.345	0.100
	CORR	0.795	0.066	0.779	0.067
B (2115)	SSIM	0.453	0.037	0.179	0.043
	NRMSE	0.102	0.030	0.253	0.066
	CORR	0.818	0.056	0.835	0.045
C (1837)	SSIM	0.431	0.036	0.158	0.037
	NRMSE	0.104	0.035	0.225	0.062
	CORR	0.818	0.060	0.845	0.046
D (626)	SSIM	0.417	0.035	0.147	0.035
	NRMSE	0.087	0.028	0.176	0.043
	CORR	0.849	0.048	0.871	0.037

**Table 6:** Quality metrics for retrieved maps of Figure 10 in the case of high and low noise.

SNR		30 dB			5 dB		
**Quality Metric**		**SSIM**	**NRMSE**	**CORR**	**SSIM**	**NRMSE**	**CORR**
Breast 1	$\epsilon^{\prime }$	0.50	0.06	0.89	0.46	0.08	0.85
	σ	0.21	0.23	0.86	0.21	0.31	0.81
Breast 2	$\epsilon^{\prime }$	0.46	0.10	0.85	0.44	0.13	0.78
	σ	0.21	0.21	0.86	0.20	0.29	0.80
Breast 3	$\epsilon^{\prime }$	0.45	0.08	0.86	0.36	0.12	0.79
	σ	0.27	0.17	0.89	0.18	0.26	0.82
Breast 4	$\epsilon^{\prime }$	0.45	0.07	0.87	0.38	0.12	0.77
	σ	0.19	0.17	0.88	0.09	0.28	0.80

## Data Availability

All the data are available prior request to the authors.

## Abbreviations

The following abbreviations are used in this manuscript:

MRI	Magnetic Resonance Imaging
SNR	Signal to Noise Ratio
EIS	Electromagnetic Inverse Scattering
CNN	Convolutional Neural Network
ANN	Artificial Neural Network
UAT	Universal Approximation Theorem
AWGN	Additive, White, Gaussian Noise
MoM	Method of Moments
FFT-CG	Fast Fourier Transform-Conjugate Gradient
NRMSE	Normalised Root mean Square Error
SSIM	Structural Similarity Index Measure
DBIM	Distorted Born Iterative Method
CSI	Contrast Source Inversion
CC-CSI	Cross Correlated-Contrast Source Inversion
